# DNAM-1 chimeric receptor-engineered NK cells: a new frontier for CAR-NK cell-based immunotherapy

**DOI:** 10.3389/fimmu.2023.1197053

**Published:** 2023-06-08

**Authors:** Loredana Cifaldi, Ombretta Melaiu, Roberto Giovannoni, Monica Benvenuto, Chiara Focaccetti, Daniela Nardozi, Giovanni Barillari, Roberto Bei

**Affiliations:** ^1^ Department of Clinical Sciences and Translational Medicine, University of Rome “Tor Vergata”, Rome, Italy; ^2^ Department of Biology, University of Pisa, Pisa, Italy; ^3^ Departmental Faculty of Medicine, Saint Camillus International University of Health and Medical Sciences, Rome, Italy

**Keywords:** CAR-NK cells, solid tumors, DNAM-1, NK cell-based immunotherapy, NK cell engineering

## Abstract

DNAM-1 is a major NK cell activating receptor and, together with NKG2D and NCRs, by binding specific ligands, strongly contributes to mediating the killing of tumor or virus-infected cells. DNAM-1 specifically recognizes PVR and Nectin-2 ligands that are expressed on some virus-infected cells and on a broad spectrum of tumor cells of both hematological and solid malignancies. So far, while NK cells engineered for different antigen chimeric receptors (CARs) or chimeric NKG2D receptor have been extensively tested in preclinical and clinical studies, the use of DNAM-1 chimeric receptor-engineered NK cells has been proposed only in our recent proof-of-concept study and deserves further development. The aim of this perspective study is to describe the rationale for using this novel tool as a new anti-cancer immunotherapy.

## Introduction

NK cells are cytotoxic lymphocytes belonging to innate immunity that, by a complex array of activating and inhibitory receptors, are tolerant versus healthy cells and can recognize and kill virus-infected and transformed cells through the release of cytolytic granules and cytotoxic cytokines (1). The peculiar ability to elicit a potent response against target cells is due to the expression by NK cells of a repertoire of activating receptors such as NKG2D, the accessory molecule DNAX (DNAM-1, CD226), and natural cytotoxicity receptors (NCRs) including NKp30, NKp44, and NKp46 (2, 3). Of note, ligands for NKG2D and DNAM-1 are poorly expressed in normal cells [proteinatlas.org, Genotype-Tissue Expression (GTEx) from The Cancer Genome Atlas (TCGA) database and (4)] and highly expressed in virus-infected and transformed cells (5, 6). Furthermore, NK cells, through the expression of FcγRIIIA (CD16) receptor, are responsible for the antibody-dependent cellular cytotoxicity (ADCC) (7), which is a crucial function in the clinical context of all immunotherapies involving monoclonal antibodies (mAb) (8).

In addition to their cytotoxic function, NK cells play a crucial role in regulating the maturation and activation state of other immune cells, through sophisticated cross-talks and biological mechanisms that further support their use in immunotherapy (9).

In contrast, it is noteworthy that NK cells in cancer patients show impaired functions accompanied by a poor ability to infiltrate the tumor microenvironment (TME), as tumor cells adopt different various immune evasion mechanisms (10–17). Therefore, the adoptive transfer of *ex vivo* expanded and activated allogeneic NK cells for immunotherapy turns out to be a strategic clinical adoption to help cancer patients to fight tumor cells, thus attracting increasing interest in the past decade (18).

Primary allogeneic and alloreactive NK cells, from healthy donors with a favorable immunoglobulin-like receptor (KIR)-human leukocyte antigen (HLA) mismatch (19), can be harvested from several sources such as peripheral blood (20), umbilical cord blood (21) or be derived by induced pluripotent stem cells (iPSC) (22, 23). Compared with the therapeutic use of T cells, that of allogeneic NK cells has several advantages: this has progressively stimulated the improvement of previously limited *ex vivo* amplification methods of NK cells and designs for the expression of various chimeric antigen receptors (CARs) and NKG2D chimeric receptor (24, 25) suitable for clinical use (ClinilTrial.gov and **Supplementary Table S1**).

In this context, one should consider that T and NK cells are often dysfunctional in cancer patients, limiting the use of autologous cells for engineered manipulation (26). Noticeably, NK cells display greater antitumor effects in allogeneic settings than in autologous ones (20, 27). However, the use of allogeneic T or CAR-T cells presents limitations related to severe haploidentical mismatch conditions necessary to reduce the risk of graft-versus-host disease (GvHD) and cytokine release syndrome (28–30). In contrast, allogeneic NK cells do not cause GvHD (31–33) and display a low risk of proliferation in transfused patients and, thus a major safety, as compared with infused T cells. Finally, the high availability of allogeneic NK cells, their low cost compared to CAR-T cells, and the possibility of cryopreserving them for further administration allowing the treatment of many patients from a single NK cell donor, entitles their clinical use for several types of cancers (34, 35).

So far, the successful use of NK cells engineered for several CARs and for NKG2D chimeric receptor in the hematological and solid tumor settings has been widely reported (ClinicalTrial.gov **Supplementary Table S1**). Based on the success of CD19-targeted CAR-T cells (36), approved by U.S. Food and Drug Administration (FDA), the first CAR-NK cells were engineered with chimeric anti-CD19 single chain fragment variable (scFv) for the cure of hematologic malignancies (21). Currently, the use of CAR- or NKG2D chimeric receptor-engineered NK cells has been extended to different type of cancers; however, the number of clinical trials evaluating their efficacy against solid tumors is far lower than against hematologic malignancies (14 versus 29, as reported in **Supplementary Table S1**). This represents a clinical gap that needs to be filled. CAR-T or CAR-NK cells have generally shown greater efficacy in hematologic malignancies than in solid tumors, mainly for the following reasons: (i) firstly, the accessibility of CAR-T or CAR-NK cells to tumor cells is significantly different between solid and hematological tumors, depending on cell morphology (absence or presence of cell-cell adhesions) and body distribution; (ii) secondly, solid tumor cells are less sensitive to cytotoxic lymphocytes, as the immune suppression mechanisms occurring in TME constitute a barrier to lymphocyte infiltration. Therefore, in order to improve the efficacy of the adoptive transfer of CAR-NK cells for immunotherapy of solid tumors, the search for more specific tumor target molecules, accompanied by mechanisms that overcome the barriers of TME, still needs to be extensively explored (37).

Aiming to fill this gap, recently we have provided promising *in vitro* results on the efficacy of never before explored DNAM-1-chimeric receptor-engineered NK cells against neuroblastoma (NB) (38). This proof-of-concept study is prompting us at optimizing the DNAM-1-based chimeric construct with the aim of developing highly efficient DNAM-1 chimeric receptor-engineered NK cells to be employed in preclinical studies and prospective clinical trials primarily directed against solid tumors.

## DNAM-1

Human DNAX accessory molecule-1 (DNAM-1, CD226) is constitutively expressed in T, NK cells, and some myeloid cells. It is a type I transmembrane glycoprotein containing a leader sequence of 18 amino acid (aa), two extracellular Ig-like C2-set domains of 230 aa, a transmembrane domain of 28 aa and a cytoplasmic region of 60 aa. Together with other activating receptors, such as NKG2D and NCRs (39), DNAM-1 triggers powerful activating signals that promote NK cell-mediated cytotoxicity and cytokine secretion (40, 41). DNAM-1 mediates activation signals through the engagement with two ligands such as PVR (poliovirus receptor, CD155) and Nectin-2 (poliovirus receptor-related 2 protein, PVRL2, also known as CD112) (5). Furthermore, through cis-binding to the integrin LFA-1 upon the engagement of LFA-1 with ICAM-1 (42), DNAM-1 undergoes phosphorylation at conserved amino acid residues in its cytoplasmic domain such as tyrosine 322 [Y322 in human and Y319 in mouse, (42)] and serine 326 (40) *via* Src family kinase Fyn and protein kinase C, respectively (43). The coordinated expression of DNAM-1 and LFA-1 is also crucial for NK cell education (44).

Adequate expression of DNAM-1 enables NK cells to recognize and kill hematopoietic malignancies such as acute myeloid leukemia (AML) (45), multiple myeloma (MM) (39), and solid tumor cells such as melanoma (46) and NB (47), thus contributing to a favorable prognosis (45, 48). In contrast, DNAM-1 expression is impaired in AML cancer patients and its loss has been correlated with the tumor severity (49).

## PVR and Nectin-2 in cancer patients

Both PVR and Nectin-2 ligands are closely linked to tumorigenesis. Indeed, in addition to being expressed in virus-infected cells (43), these ligands are overexpressed in several hematological and solid tumors (5, 50–52). Noticeably, these ligands, in particular PVR, are potential prognostic markers in AML (53, 54), MM (55), hepatocellular carcinoma (56), and bladder urothelial carcinoma (BLCA) (57). As we have previously reported, PVR expression is directly under the control of p53 at promoter level (47), whilst the transcriptional regulation of Nectin-2 remains more widely to be explored (58). Furthermore, PVR and Nectin-2 are both upregulated by Toll-like receptors agonists in dendritic cells (59, 60) and by DNA-damage response in multiple myeloma cells (61) or in Ag-activated T lymphocytes (62). In addition, PVR is upregulated by IFN-γ in NB cell lines (63) and epigenetic modulations in malignant lymphocytes (64), while it is downregulated by the human immunodeficiency virus type 1 Nef and Vpu proteins (65) and the human cytomegalovirus UL141 protein (66).

The activating signal mediated by DNAM-1 following the engagement of the ligands PVR or Nectin-2 is counteracted by the competing binding of inhibitory receptors such as TIGIT (T-cell immunoglobulin and ITIM domain) (67), TACTILE (T cell activation, increased late expression, also known as CD96) (68) and PVRIG (69) for the same ligands. In particular, PVR is recognized by TIGIT and TACTILE (70, 71), while Nectin-2 is recognized by TIGIT and PVRIG (69, 70). For this reason, TIGIT, TACTILE and PVRIG have been considered targets for checkpoint blockade immunotherapy (72). Of note, the high expression levels of PVR, typical of various tumor types, revealed its hypothetical proto-oncogenic role, leading researchers to develop therapeutic strategies that directly target PVR (73).

## DNAM-1 chimeric receptor-engineered NK cells

Adoptive transfer of activated NK cells expressing higher and more stable levels of DNAM-1, might be a useful clinical approach to help cancer patients to fight tumor cells. The DNAM-1 chimeric receptor could confer a dual advantage to NK cells: (i) specific recognition of ligands such as PVR and Nectin-2, which are highly expressed in tumor cells, but importantly absent or poorly expressed in normal cells, and (ii) its overexpression, which should result in a favorable molecular imbalance with respect to the normal expression of competing receptors (TIGIT, TACTILE, PVRIG), leading to its increased binding to PVR and Nectin-2. In addition, its function could be strategically improved by in-frame expression of costimulatory molecules that support cytotoxic activity and overcome TME immune escape mechanisms. We previously reported a proof-of-concept study on the activity of DNAM-1-chimeric receptor-engineered NK cells obtained by transient transfection of primary human NK cells for a DNAM-1-chimeric receptor (38). Specifically, we compared four different constructs, including the full-length DNAM-1 receptor, and three different DNAM-1-based chimeric receptors providing the expression of DNAM-1 in frame with costimulatory molecules such as 2B4 and CD3ζ, and we showed that the DNAM-1-CD3ζ construct, which recapitulates a first generation of DNAM-1 chimeric receptor, yielded the best results in terms of expression of DNAM-1 chimeric receptor and NK cell functions. Furthermore, DNAM-1-CD3ζ engineered NK cells were particularly more effective to recognize and kill two NB cell lines, LAN-5 and SMS-KCNR, treated with Nutlin-3a, an MDM2 targeting drug with immunomodulatory effects on the upregulation of ligands for NK cell-activating receptors, including PVR and Nectin-2 (47). Therefore, the combined use of DNAM-1-CD3ζ engineered NK cells with Nutlin-3a in tumors that retain p53-wt, such as most forms of NB, with the exception of some cases of relapse (74), may represent a novel therapeutic approach for solid tumors.

## *In-silico* analysis of PVR and Nectin-2 in solid tumor patients

**Figure 1:**
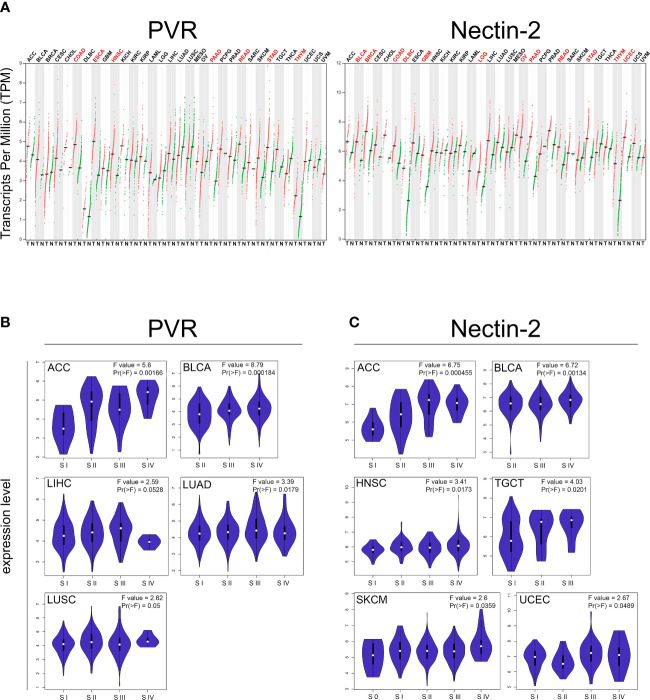
*In-silico* bioinformatics analysis of *PVR* and *NECTIN2* gene expression by GEPIA2 web-tool based on The Cancer Genome Atlas (TCGA) database. **(A)** Dot plot profiling of PVR (top) and Nectin-2 (down) differential expression levels in 33 cancer types, derived from TCGA database, compared to the normal, derived from TCGA or Genotype-Tissue Expression (GTEx). Each dot represents a distinct tumor (red) or normal sample (green) while each column represents a different tumor type (tumor labels and sample sizes are reported in **Supplementary Table 2**). The transcript per million (TPM) value, shown in ordinate, is used to display the relative gene expression. Tumor labels are indicated in red when there is a significant difference between tumor (T) versus normal (N) tissues. Data were analyzed by ANOVA test. |log2FC| > 1 and FDR < 0.05 were considered as differentially expressed. **(B, C)** Violin plots showing the expression level of PVR **(B)** and Nectin-2 **(C)** among different pathologic stages (S) of indicated solid tumors. F-value indicates the statistical value of the F test; Pr (> F) indicates *p* value. A *p* value of < 0.05 was considered statistically significant.

The widely reported high expression of both PVR and Nectin-2 in solid tumor cells and very low expression in normal cells [protein.atlas.gov and GTEx from TCGA database], was the main reason for choosing to engineer NK cells with a DNAM-1 chimeric receptor. In order to further explore the expression of both PVR and Nectin-2 in solid tumors, and to prospectively propose the adoptive transfer of DNAM-1 chimeric receptor-engineered NK cells also in adult solid malignancies, we performed an *in-silico* bioinformatic analysis by using GEPIA2 (www.gepia2.cancer-pku.cn, **Figure 1**). Specifically, we queried this online tool providing data concerning gene expression and tumor stage/grade, to compare the expression of selected genes between tumor and normal tissues, based on TCGA. Interestingly, we found that the expression profile of both PVR and Nectin-2 resulted higher in several tumor samples than in paired normal tissues across a broad spectrum of solid tumors. In particular, the expression of PVR was significantly higher in colon adenocarcinoma (COAD), esophageal carcinoma (ESCA), head and neck squamous cell carcinoma (HNSC), pancreatic adenocarcinoma (PAAD), rectum adenocarcinoma (READ), stomach adenocarcinoma (STAD) and thymoma (THYM), while that of Nectin-2 was significantly higher in bladder urothelial carcinoma (BLCA), breast invasive carcinoma (BRCA), COAD, lymphoid neoplasm diffuse large B-cell lymphoma (DLBC), glioblastoma multiforme (GBM), brain lower grade glioma (LGG), ovarian serous cystadenocarcinoma (OV), PAAD, READ, STAD, THYM and uterine corpus endometrial carcinoma (UCEC) (**Figure 1A**). In addition, the higher expression of PVR or Nectin-2 correlated with the advanced stage of different forms of solid tumors. In particular, PVR higher expression correlated with the advanced stage of adrenocortical carcinoma (ACC), BLCA, liver hepatocellular carcinoma (LIHC), lung adenocarcinoma (LUAD), lung squamous cell carcinoma (LUSC) (**Figure 1B**), while that of Nectin-2 correlated with the advanced stage of ACC, BLCA, HNSC, testicular germ cell tumors (TGCT), skin cutaneous melanoma (SKCM) and UCEC (**Figure 1C**). These data indicate that the high expression of PVR and Nectin-2 in tumor cells compared to normal cells affects several solid tumors, supporting the hypothesis of a wide prospective clinical use of DNAM-1 chimeric receptor-engineered NK cells.

Furthermore, we used the R2 Genomics Analysis and Visualization Platform (https://hgserver1.amc.nl/cgi-bin/r2/main.cgi?open_page=login) to investigate the prognostic value of PVR and Nectin-2 ligands in a variety of tumor types. We found that higher expression of PVR significantly correlated with lower patient overall survival in ACC, BLCA, COAD, ESCA, HNSC, kidney renal clear cell carcinoma (KIRC), kidney renal papillary cell carcinoma (KIRP), LUAD, LUSC, mesothelioma (MESO), OV, prostate adenocarcinoma (PRAD), SKCM, STAD and uveal melanoma (UVM) (**Supplementary Figure 1A**). By contrast, the lower expression of PVR significantly correlated with lower patient survival in BRCA, PAAD, READ and THYM (**Supplementary Figure 1B**), in agreement with published data from a cohort of patients with a pediatric form of solid tumor such as NB (75). Similarly, the higher expression of Nectin-2 correlated with lower patient overall survival in KIRC, KIRP, GBM, HNSC, LIHC, LUAD, LUSC, MESO, OV, READ, SKCM, UCEC and uterine carcinosarcoma (UCS) (**Supplementary Figure 2A**). By contrast, the lower expression of Nectin-2 correlated with lower patient overall survival in BRCA, COAD, ESCA, PRAD, STAD and UVM (**Supplementary Figure 2B**). These data suggest that the expression levels of both PVR and Nectin-2 can correlate differently with patient overall survival, depending on the kind of solid tumors.

## Clinical perspective

With a view to finding an optimized off-the-shelf product for cellular immunotherapeutic approaches, we foresee that DNAM-1 chimeric receptor engineered-NK cells have several strengths that should be taken into account. NK cells engineered for a chimeric form of an activating receptor such as DNAM-1 are likely to specifically target tumor cells which express high levels of PVR and Nectin-2 (**Figure 1**), while should be tolerant of normal cells expressing low levels of PVR and Nectin-2 [protein.atlas.org, GTEx from TCGA database and (4)]. This represents an advantage over many types of single-chain antibody-based CAR-engineered lymphocytes designed to target proteins expressed not only by tumor cells but also, at high physiological levels, by various normal cells such as CD19 and B220 (B lymphocytes and follicular dendritic cells), disialoganglioside or GD2 (neurons, skin melanocytes and peripheral nerves), human epidermal growth factor receptor 2 or HER2 (many tissues), prostate-specific membrane antigen or PSMA (kidneys, small intestine and salivary glands), etc. This non-selective tumor specificity is often the cause of high toxicity and adverse effects due to the cytotoxic reaction mediated by CAR-lymphocytes against normal tissues. So far, with a restricted expression in normal tissues and overexpression in many types of solid tumors, B7-H3 resulted a more promising therapeutic target compared to the others (76). DNAM-1 ligands PVR and Nectin-2 have been described to be absent or very scarcely expressed in normal tissue [proteinatlas.org and (73, 77)], so their targeting should hypothetically not be toxic; however, the differential expression of DNAM-1 ligands in cancer versus normal cells does not exclude a possible toxicity mediated by DNAM-1 chimeric receptor-engineered NK cells, which should be carefully explored by preclinical studies.

**Figure 2:**
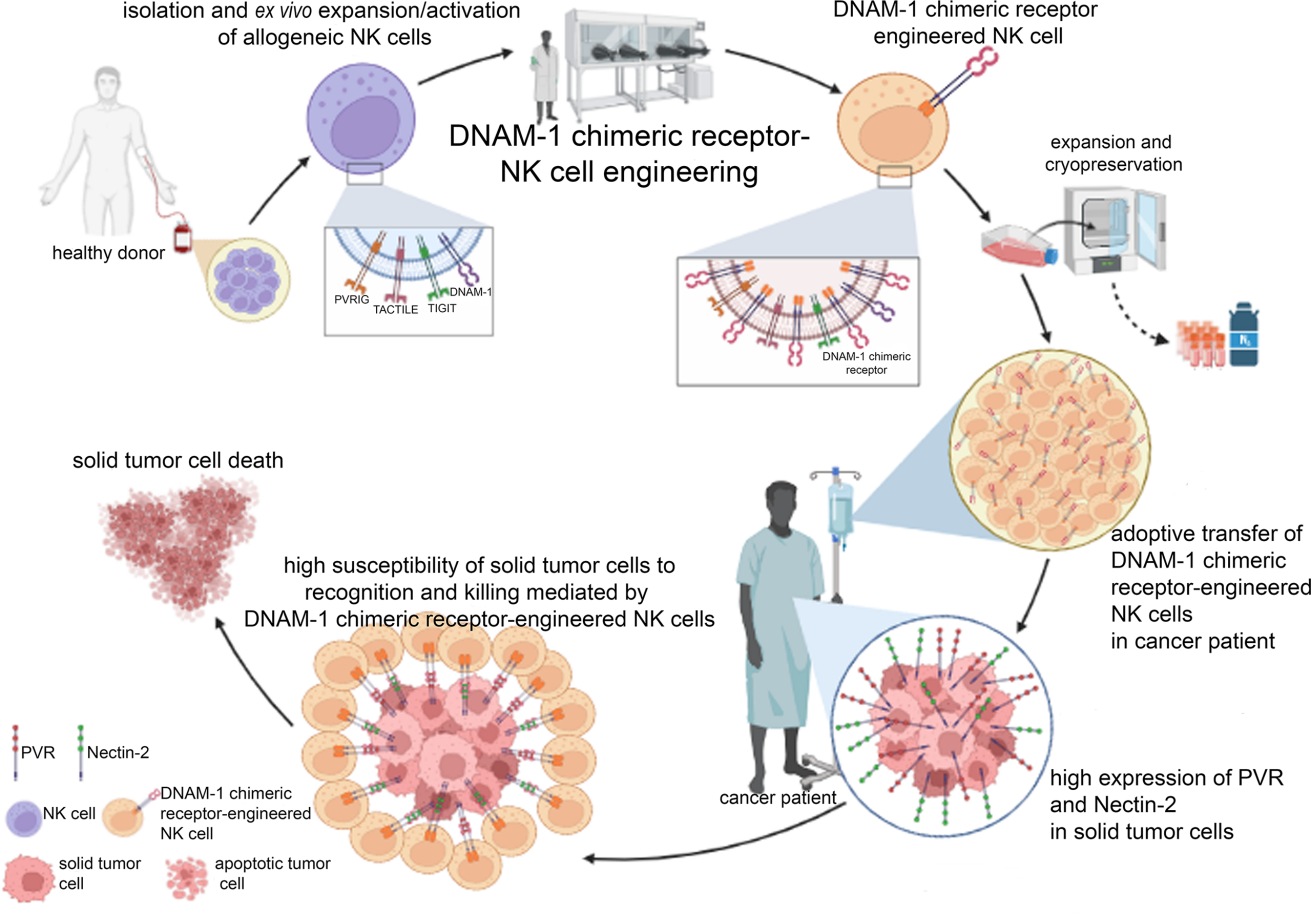
Clinical perspective of the GMP manufacturing and clinical use of DNAM-1 chimeric receptor-engineered NK cells. After leukapheresis of a healthy HLA-related donor, mature alloreactive NK cells can be isolated to be firstly *ex vivo* expanded and activated and then engineered for the expression of DNAM-1 chimeric receptor. Large quantities of DNAM-1 chimeric receptor-engineered NK cells can be obtained to be infused in cancer patient or cryopreserved for future use. The high expression of PVR and Nectin-2 specifically in tumor cells should facilitate their recognition mainly by DNAM-1 chimeric receptor compared to competing receptors (TIGIT, TACTILE and PVRIG), thus promoting tumor cell death. The figure was created with Biorender (https://biorender.com/).

For a hypothetic good manufacturing practice (GMP) production and clinical use of DNAM-1 chimeric receptor-engineered NK cells, primary NK cells should be isolated through leukapheresis by the blood of a HLA-matched unrelated healthy donor, *ex vivo* expanded and activated, engineered for the expression of DNAM-1 chimeric receptor, expanded to be infused in cancer patients or be cryopreserved for future use (**Figure 2**). Different modes of administration should be considered, depending on the type and location of the tumor in the body, such as intravenous or local injection. DNAM-1 chimeric receptor, expressed at stable and high levels, should strongly compete for the binding of PVR and Nectin-2 with the agonist receptors TIGIT, TACTILE and PVRIG, thus favoring activating cytotoxic signals over inhibitory ones. The high expression of PVR and Nectin-2 in tumor cells could make them strongly susceptible to DNAM-1 chimeric receptor-engineered NK cell-mediated recognition and killing. Within days after the injection of DNAM-1 chimeric receptor-engineered NK cells, tumor cell death could occur at the tumor site and lead the patient to an objective clinical response, depending on the aggressiveness and size of primary or secondary tumor masses. To avoid recurrence, the number of administrations of DNAM-1 chimeric receptor-engineered NK cells should be carefully planned, depending on the characteristics of the tumor, such as location, extent, stage, or presence of metastasis. To enhance the anticancer efficiency, the use of DNAM-1 chimeric receptor-engineered NK cells could be combined with that of current anticancer cytotoxic drugs (78, 79), activating cytokines or mAbs recognizing immune checkpoint molecules (80). Ideally, the administration of DNAM-1 chimeric receptor-engineered NK cells should be also considered after surgical removal of solid tumor masses to avoid the risk of developing the minimal residual disease (MRD).

## Conclusion

The adoptive transfer of DNAM-1 chimeric receptor-engineered NK cells is expected to represent an innovative strategic clinical tool to help cancer patients in fighting solid tumors. Therefore, the development of preclinical and clinical studies aimed at obtaining stable, nontoxic, highly antitumor cytotoxic DNAM-1 chimeric receptor-engineered NK cells, in high quantities for cryopreservation and immediate future use, applicable to a broad spectrum of solid tumors, deserves further exploration.

## Data availability statement

The original contributions presented in the study are included in the article/**Supplementary Materials**, further inquiries can be directed to the corresponding author/s.

## Author contributions

All authors listed have made a substantial, direct, and intellectual contribution to the work and approved it for publication.

## Supplementary material

Supplementary Figure 1 Overall survival probability of patients with the indicated solid tumor type (tumor labelling is explained in **Supplementary Table 2**) in each graph carrying high (blue line) or low (red line) *PVR* gene expression. High *PVR* gene expression can correlate with a worse (A) or favorable overall survival (B). Statistically significant *p* values are indicated.

Supplementary Figure 2 Overall survival probability of patients with the indicated solid tumor type (tumor labelling is explained in **Supplementary Table 2** in each graph carrying high (blue line) or low (red line) *NECTIN2* gene expression. High *NECTIN2* gene expression can correlate with a worse (A) or favorable overall survival (B). Statistically significant *p* values are indicated.
