# Supplementary figures and images for: DNAM-1 chimeric receptor-engineered NK cells: a new frontier for CAR-NK cell-based immunotherapy
Source: Front Immunol. 2023 Jun 8;14:1197053. doi: 10.3389/fimmu.2023.1197053 (PMC10285446; doi:10.3389/fimmu.2023.1197053)

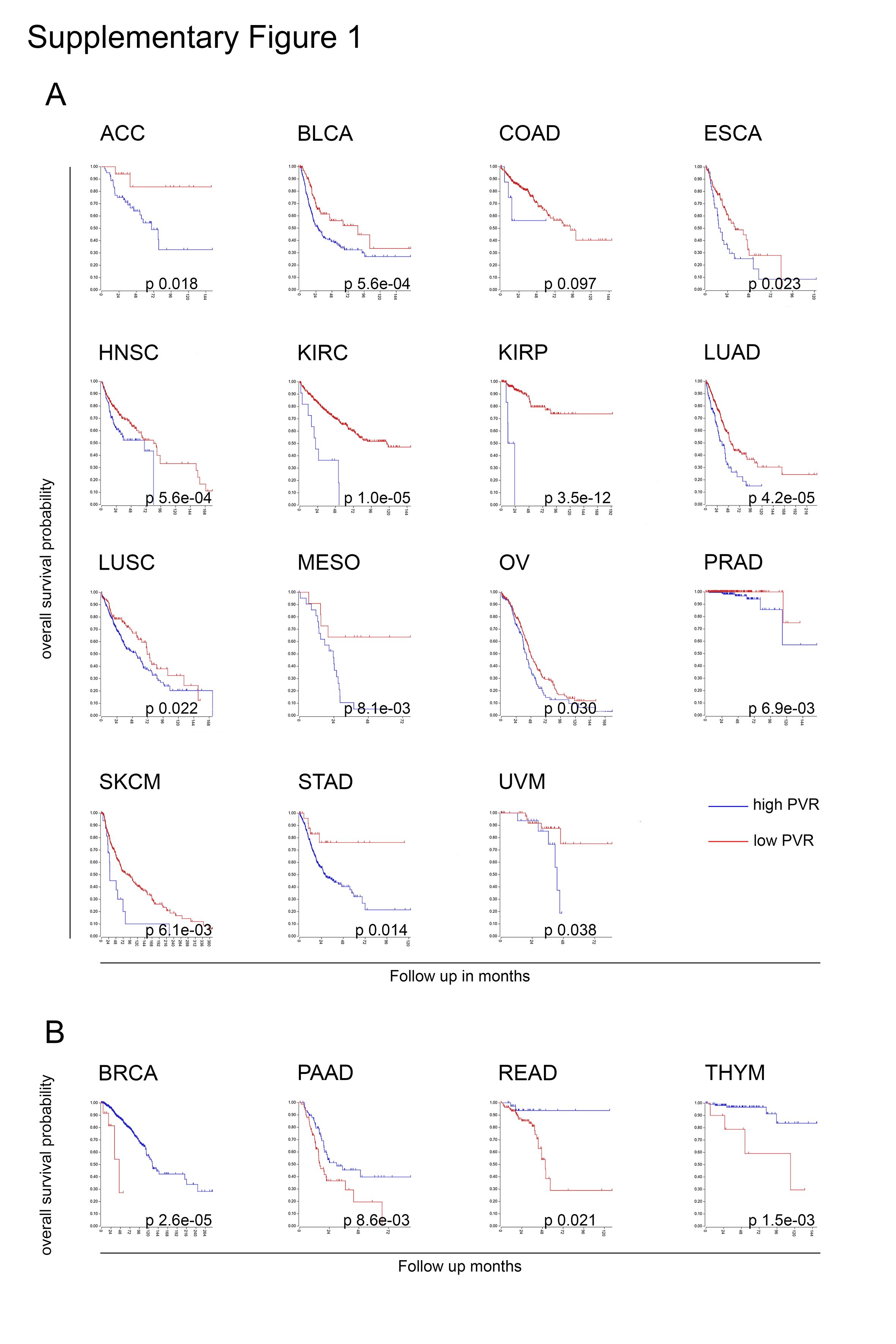

Supplement: Supplementary Figure 1 — Overall survival probability of patients with the indicated solid tumor type (tumor labelling is explained in Supplementary Table 2 ) in each graph carrying high (blue line) or low (red line) PVR gene expression. High PVR gene expression can correlate with a worse (A) or favorable overall survival (B). Statistically significant p values are indicated. [file Image_1.tiff]

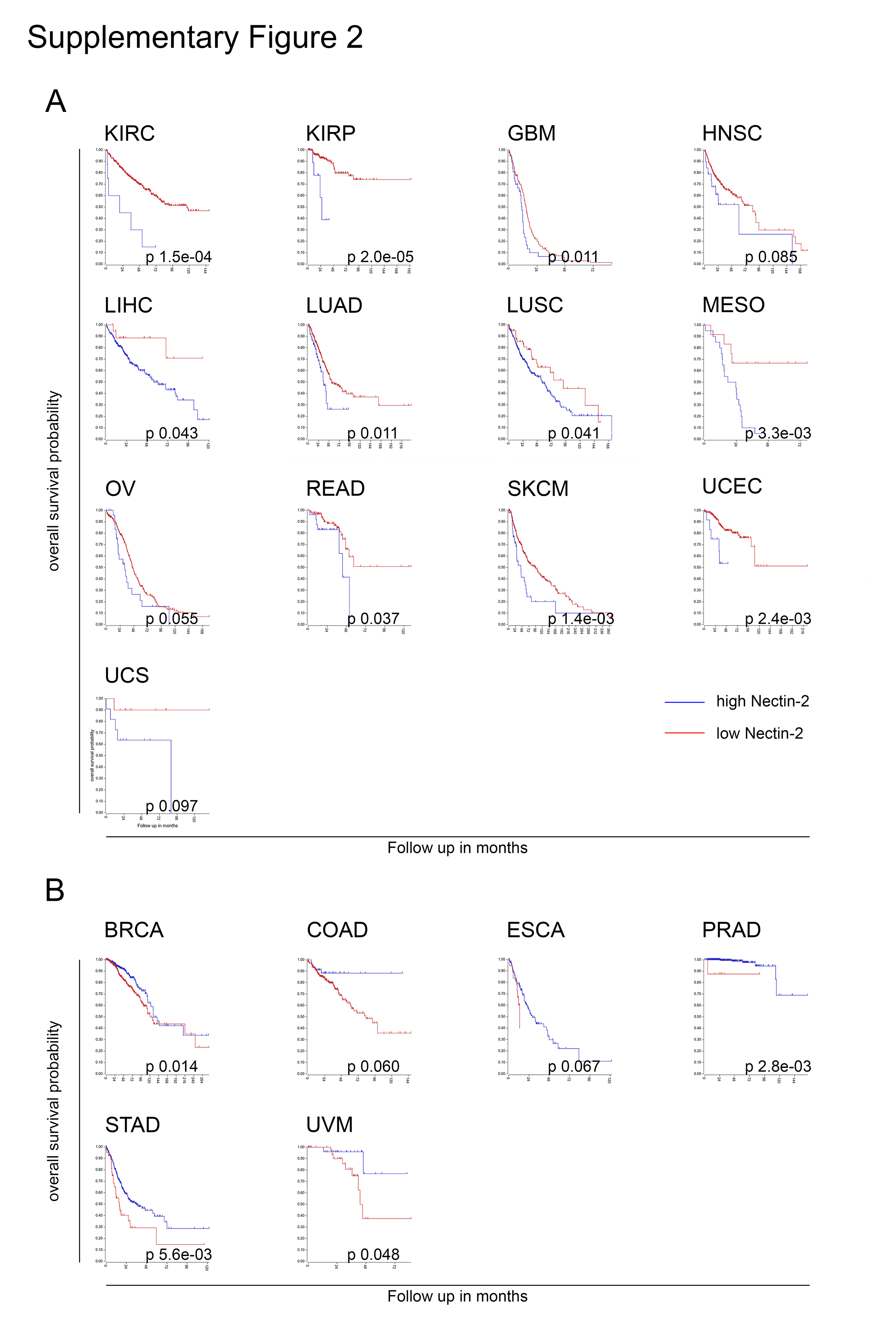

Supplement: Supplementary Figure 2 — Overall survival probability of patients with the indicated solid tumor type (tumor labelling is explained in Supplementary Table 2 in each graph carrying high (blue line) or low (red line) NECTIN2 gene expression. High NECTIN2 gene expression can correlate with a worse (A) or favorable overall survival (B). Statistically significant p values are indicated. [file Image_2.tiff]
