# Supplementary material for: DNAM-1 chimeric receptor-engineered NK cells: a new frontier for CAR-NK cell-based immunotherapy
Source: Front Immunol. 2023 Jun 8;14:1197053. doi: 10.3389/fimmu.2023.1197053 (PMC10285446; doi:10.3389/fimmu.2023.1197053)
Supplement: Supplementary file 3 [file Table_1.docx]

| **Supplementary Table 1**  Clinical trials in recruiting, completed and terminated status on immunotherapies based on the adoptive transfer of CAR- and NKG2D chimeric receptor engineered-NK cells, alone or in combination with chemotherapeutic drugs, monoclonal antibodies (mAbs), immune checkpoint inhibitors or cytokines. | | | | | |
| --- | --- | --- | --- | --- | --- |
| **CAR-NK cells** | | | | | |
| *mAb* | *in combination with* | *Clinical Trial identifier* | *phase* | *status* | *Tumor* |
| α-CD19 | - | NCT05739227 | Early I | recruiting | ALL, BCC |
|  | (/CD70) | NCT05667155 | I | recruiting | BCNHL |
|  | -  - | NCT04887012 (HLA haploidentical)  NCT05472558 (Cord Blood-derived) | I  I | recruiting  recruiting | BCNHL |
|  | - | NCT03056339 | I and II | completed | RR-BLM |
|  | - | NCT05472558 | I | recruiting | BCNHL |
|  | rituximab, cyclophosphamide  (iCasp9/IL-15) | NCT05379647 | I | recruiting | RR-BLM |
|  | - | NCT05410041 | I | recruiting | BCNHL, ALL, AML |
|  | fludarabine, cyclophosphamide | NCT04796675 | I | recruiting | BCNHL, ALL, AML |
|  | - | NCT05654038 | I and II | recruiting | BCC |
|  | - | NCT05563545 | I | completed | AML |
|  | - | NCT05645601 | I | recruiting | RR-BLM |
|  | fludarabine, cyclophosphamide | NCT04796688 | I | recruiting | B-ALL, B-CLL |
|  | fludarabine, cyclophosphamide | NCT05020015 | II | recruiting | BCNHL |
|  | IL-2, lymphodepleting chemotherapy | NCT05336409 | I | recruiting | RR-BLM |
|  | (NKX019, /mbIL-15) | NCT05020678 | I | recruiting | HM |
| α-CD70/IL-15 | fludarabine, cyclophosphamide | NCT05092451 | I and II | recruiting | AML, LY, MM |
|  |  | NCT05703854 | I and II | Not yet recruiting | ARCC, AM, AO |
| α-CD276 | - | NCT05143151 | I and II | recruiting | APC |
| α-DLL3 | - | NCT05507593 | I | recruiting | SCLC |
| α-CD123 | - | NCT05574608 | I | recruiting | RR-AML |
| α-CD5 | fludarabine, cyclophosphamide (/IL-15, cord blood NK cells) | NCT05110742 | I and II | recruiting | HM |
| α-5T4 | - | NCT05194709 | Early I | recruiting | AST |
|  | - | NCT05137275 | Early I | recruiting | AST |
| α-claudin6 | - | NCT05410717 | I and II | recruiting | AST |
| α-PD-L1 | pembrolizumab | NCT04847466 | II | recruiting | GC, HNC |
| α-GD2 (NKT) | - | NCT05650749 | I | Not yet recruiting | NB |
| α-BCMA | - | NCT05008536 | I | recruiting | R-MM |
|  | fludarabine, cyclophosphamide, daratumumab | NCT05182073 | I | recruiting | MM |
|  |  | NCT05652530 | I | recruiting | MM |
| α-CD33 | fludarabine, cyclophosphamide | NCT05665075 | I | recruiting | AML |
|  |  | NCT05601466 | I | recruiting | AML |
|  | fludarabine, cytoxan | NCT05008575 | I | recruiting | AML |
|  | (/α-CLL1) | NCT05215015 | I | recruiting | AML |
| α-PSMA | fludarabine, cyclophosphamide | NCT03692663 | I | recruiting | MCR-PC |
| α -HER2 | (NK-92) | NCT03383978 | I | recruiting | G |
|  | fludarabine, cyclophosphamide | NCT04319757 | I | recruiting | HER2-ST |
| **Chimeric NKG2D activating receptor** | | | | | |
| chimeric NKG2D | lymphodepleting chemotherapy | NCT05776355 | NA | recruiting | OC |
|  | lymphodepleting chemotherapy | NCT05734898 | NA | recruiting | AML |
|  | - | NCT05247957 | I | terminated | RR-AML |
|  | - | NCT05213195 | I | recruiting | M-CC |
|  | (NK-92) | NCT05528341 | I | recruiting | RR-ST |
|  | (NKX101, /mbIL-15) | NCT04623944 | I | recruiting | HM |

Abbreviation: ALL, Acute Lymphoblastic Leukemia; AML, Acute Myeloid Leukemia; APC, Advanced Pancreatic Carcinoma; AST, Advanced Solid Tumors; ARCC, Advanced Renal Cell Carcinoma; AM, Advanced Mesothelioma; AO, Advanced Osteosarcoma; B-CLL, B-cell Chronic Lymphoblastic Leukemia; B-LL, B-cell Lymphoblastic Leukemia; BCC, B-cell cancers; BCNHL, B-cell non-Hodgkin’s lymphoma; G, Glioblastoma; GC, Gastric cancer; HER2-ST, HER-2 expressing Solid Tumor; HM, Hematological Malignancies; HNC, Head and Neck cancer; LY, Lymphoma; M-CC, Metastatic Colorectal cancer; MCR-PC, Metastatic Castration Resistant Prostate Cancer; MM, Multiple Mieloma; NB, Neuroblastoma; R-MM, Refractory Multiple Mieloma; RR-AML, Relapsed/Refractory Acute Myeloid Leukemia; RR-BLM, Relapsed/Refractory B-Lymphoid Malignancies; RR-ST, Relapsed/Refractory Solid Tumors; SCLC, Small Cell Lung Cancer; OC, Ovarian Cancer.

Daratumumab, α-CD38; Pembrolizumab, α-PD-1; Rituximab, α-CD20.

Fludarabine, cyclophosphamide, Cytoxan: chemotherapeutic/lymphodepletion drugs.

In () are reported, were available, co-expressed chimeric molecules or NK cell source (cord blood, NK-92 cell line).

NA, not applicable
