# Supplementary material for: DNAM-1 chimeric receptor-engineered NK cells: a new frontier for CAR-NK cell-based immunotherapy
Source: Front Immunol. 2023 Jun 8;14:1197053. doi: 10.3389/fimmu.2023.1197053 (PMC10285446; doi:10.3389/fimmu.2023.1197053)
Supplement: Supplementary file 4 [file Table_2.docx]

| **Supplementary Table 2**  List of solid tumors labels, details of their relative solid tumor type and number of tumor and normal tissue samples provided by TCGA and GTEx, used for *in-silico* GEPIA2 bioinformatics analysis, shown in Figure 1. Details of solid tumor type are valid also for tumor labels shown in both Supplementary Figures 1 and 2. | | | | | |
| --- | --- | --- | --- | --- | --- |
| **label** | **solid tumor type** | **TCGA** | **normal tissue** | **TCGA** | **GTEx** |
| ACC | Adrenocortical carcinoma | 77 | Adrenal Gland | - | 128 |
| BLCA | Bladder Urothelial Carcinoma | 404 | Bladder | 19 | 9 |
| BRCA | Breast invasive carcinoma | 1085 | Breast | 112 | 179 |
| CESC | Cervical squamous cell carcinoma and endocervical adenocarcinoma | 306 | Cervix Uteri | 3 | 10 |
| CHOL | Cholangio carcinoma | 36 | - | 9 | - |
| COAD | Colon adenocarcinoma | 275 | Colon | 41 | 308 |
| DLBC | Lymphoid Neoplasm Diffuse Large B-cell Lymphoma | 47 | Blood | - | 337 |
| ESCA | Esophageal carcinoma | 182 | Esophagus | 13 | 273 |
| GBM | Glioblastoma multiforme | 163 | Brain | - | 207 |
| HNSC | Head and Neck squamous cell carcinoma | 519 | - | 44 | - |
| KICH | Kidney Chromophobe | 66 | Kidney | 25 | 28 |
| KIRC | Kidney renal clear cell carcinoma | 523 | Kidney | 72 | 28 |
| KIRP | Kidney renal papillary cell carcinoma | 286 | Kidney | 32 | 28 |
| LAML | Acute Myeloid Leukemia | 173 | Bone Marrow | - | 70 |
| LGG | Brain Lower Grade Glioma | 518 | Brain | - | 207 |
| LIHC | Liver hepatocellular carcinoma | 369 | Liver | 50 | 110 |
| LUAD | Lung adenocarcinoma | 483 | Lung | 59 | 288 |
| LUSC | Lung squamous cell carcinoma | 486 | Lung | 50 | 288 |
| MESO | Mesothelioma | 87 | - | - | - |
| OV | Ovarian serous cystadenocarcinoma | 426 | Ovary | - | 88 |
| PAAD | Pancreatic adenocarcinoma | 179 | Pancreas | 4 | 167 |
| PCPG | Pheochromocytoma and Paraganglioma | 182 | - | 3 | - |
| PRAD | Prostate adenocarcinoma | 492 | Prostate | 52 | 100 |
| READ | Rectum adenocarcinoma | 92 | Colon | 10 | 308 |
| SARC | Sarcoma | 262 | - | 2 | - |
| SKCM | Skin Cutaneous Melanoma | 461 | Skin | 1 | 557 |
| STAD | Stomach adenocarcinoma | 408 | Stomach | 36 | 175 |
| TGCT | Testicular Germ Cell Tumors | 137 | Testis | - | 165 |
| THCA | Thyroid carcinoma | 512 | Thyroid | 59 | 278 |
| THYM | Thymoma | 118 | Blood | 2 | 337 |
| UCEC | Uterine Corpus Endometrial Carcinoma | 174 | Uterus | 13 | 78 |
| UCS | Uterine Carcinosarcoma | 57 | Uterus | - | 78 |
| UVM | Uveal Melanoma | 79 | - | - | - |
